# Face to Face With Renal Cell Carcinoma: A Case Report

**DOI:** 10.7759/cureus.81469

**Published:** 2025-03-30

**Authors:** Mariana A Andrade, Filipa Caires, Carla C Duarte, Catarina Santana, Cristina Saldanha

**Affiliations:** 1 Caniço Health Center, Hospital Dr. Nélio Mendonça, Serviço de Saúde da Região Autónoma da Madeira (SESARAM), Funchal, PRT; 2 Machico Health Center, Hospital Dr. Nélio Mendonça, Serviço de Saúde da Região Autónoma da Madeira (SESARAM), Funchal, PRT

**Keywords:** family doctor, kidney neoplasm, metastasis, renal cell carcinoma, world organization of family doctors

## Abstract

Renal cell carcinoma (RCC) is responsible for most cases of primary kidney neoplasms, many of which are asymptomatic until an advanced stage. According to the World Organization of Family Doctors (WONCA), in primary health care, family doctors (FD) hold a privileged position in the early diagnosis of the disease's natural history. This case presents a 61-year-old man who sought consultation with his FD for swelling in the right frontal region with a two-week history. He denied trauma or accompanying symptoms. Clinically, he presented with a right frontal swelling, 3 cm in diameter, tender to palpation, and without inflammatory signs. Given these findings, the FD requested an ultrasound of the soft tissues and later a contrast-enhanced cranial-encephalic computed tomography (CT) scan, which showed the presence of a solid nodule located in the right frontal region, centered on the bone calotte and highly vascularized, with the most likely diagnostic hypothesis being a tumor lesion, primary versus secondary. Subsequently, the patient underwent a contrast-enhanced chest-abdomen-pelvis CT, which revealed the presence of a neoplasm in the right kidney, consistent with RCC. This clinical case report aims to highlight the crucial role of the FD in detecting pathologies such as RCC, which can present in an undifferentiated manner at an early stage of its natural history, in addition to the management of preventive care and follow-up of individualized comorbidities.

## Introduction

Renal cell carcinoma (RCC) accounts for more than 90% of cases of primary kidney neoplasms, making it the most common type of urogenital cancer [1,2]. It is also the most lethal urogenital neoplasm, with a mortality rate of 30-40% [1]. It primarily affects older adults between the ages of 60 and 70 and is approximately twice as common in men [1-4]. It is also more frequent in developed countries [1,5]. Many cases are asymptomatic until an advanced stage [1,2]. At presentation, about 20-30% of patients have metastasis [1,2,4]. The incidence of RCC is increasing, mainly due to accidental diagnosis through imaging exams ordered for other reasons [1-5]. The classic triad, consisting of flank pain, hematuria, and a palpable abdominal mass, occurs in only 4-17% of cases [1,2,6] and, when present, suggests locally advanced disease [2,3]. Symptomatic patients with suggestive signs or findings should undergo further evaluation with laboratory tests (including a complete blood count, calcium levels, lactate dehydrogenase, alkaline phosphatase, C-reactive protein, liver, thyroid, and kidney function tests, and urinalysis) and abdominal computed tomography (CT) (possibly abdominal ultrasound) [1,2]. Magnetic resonance imaging (MRI) may be reserved for patients with contraindications for CT or when findings have not been fully characterized [1,2].

Since RCC is characterized by having a subtle clinical presentation, this case report aims to raise awareness of the need for high diagnostic suspicion. The only manifestation in this patient occurred through metastasis, with a differential diagnosis including benign causes in the population such as sebaceous cyst or lipoma.

## Case presentation

The patient was a 61-year-old male of Caucasian race, a businessman, married, and part of a blended family, where he lived in a house with his wife, two stepchildren, and a daughter-in-law.

His past medical history included hypertension, asthma-chronic obstructive pulmonary disease overlap syndrome, gastroesophageal reflux disease, glaucoma, initial insomnia, and obesity. He was a former smoker (pack-year history of 129). Chronically medicated with Nebivolol 5 mg, fluticasone furoate 92 μg + umeclidinium bromide 55 μg + vilanterol 22 μg, budesonide + formoterol 160 μg as needed, omeprazole 20 mg, bimatoprost 0.3 mg/ml + Timolol 5 mg/ml, dorzolamide 20 mg/ml, and bromazepam 3 mg. He had no known drug allergies.

His family medical history included his father being diagnosed with prostate cancer at 81 years old, his mother diagnosed with a brain tumor (unspecified), brother diagnosed with colon cancer at 50 years old. He was unaware of any hereditary, transmitted diseases in the family.

He presented to the clinic for an unscheduled consultation with his FD in March 2022, complaining of swelling in the right frontal region with a two-week history. There was no associated trauma or other accompanying symptoms. Upon physical examination, a 3 cm diameter swelling was observed in the right frontal region, slightly tender to palpation and without inflammatory signs. The FD ordered routine laboratory tests and an ultrasound of the soft tissues in the frontal area and scheduled a follow-up consultation. The primary differential diagnoses considered then were lipoma and sebaceous cyst in the frontal region because it was a small swelling without inflammatory signs.

About a month later, at the follow-up consultation, the laboratory results showed no significant abnormalities. The ultrasound confirmed the presence of a solid nodule in the right frontal region, centered on the bone calotte, measuring 27x18 mm, highly vascularized, with possible differential diagnoses of invasive meningioma, primary bone lesion, or metastasis.

**Figure 1:**
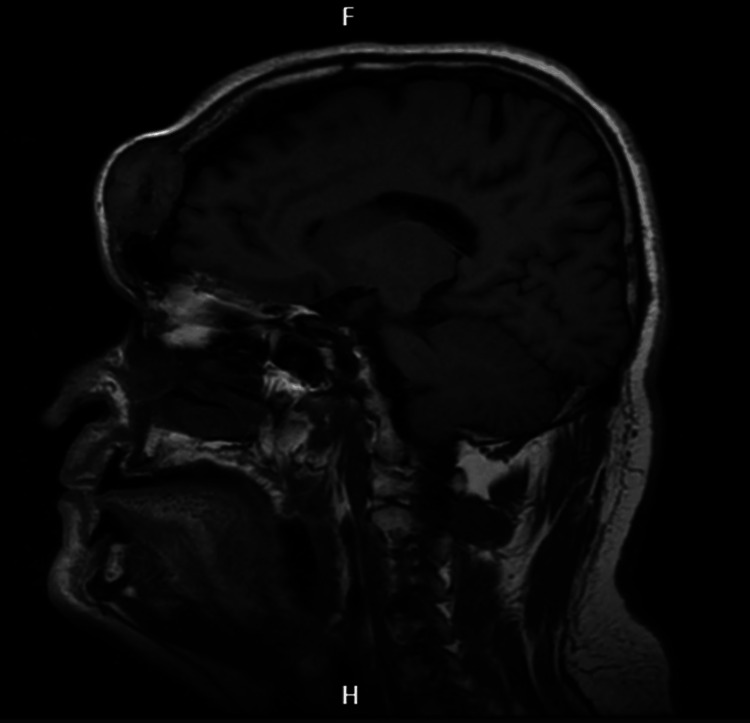
Cranioencephalic MRI in sagittal section The image shows a sagittal section in T1 fluid-attenuated inversion recovery (FLAIR).

**Figure 2:**
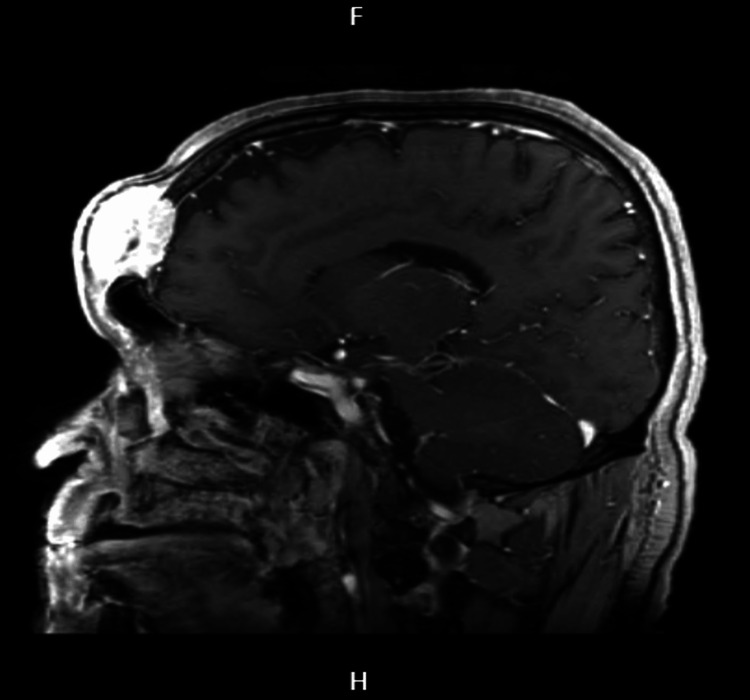
Cranioencephalic MRI in sagittal section The image shows a sagittal section in T1 gadolinium.

**Figure 3:**
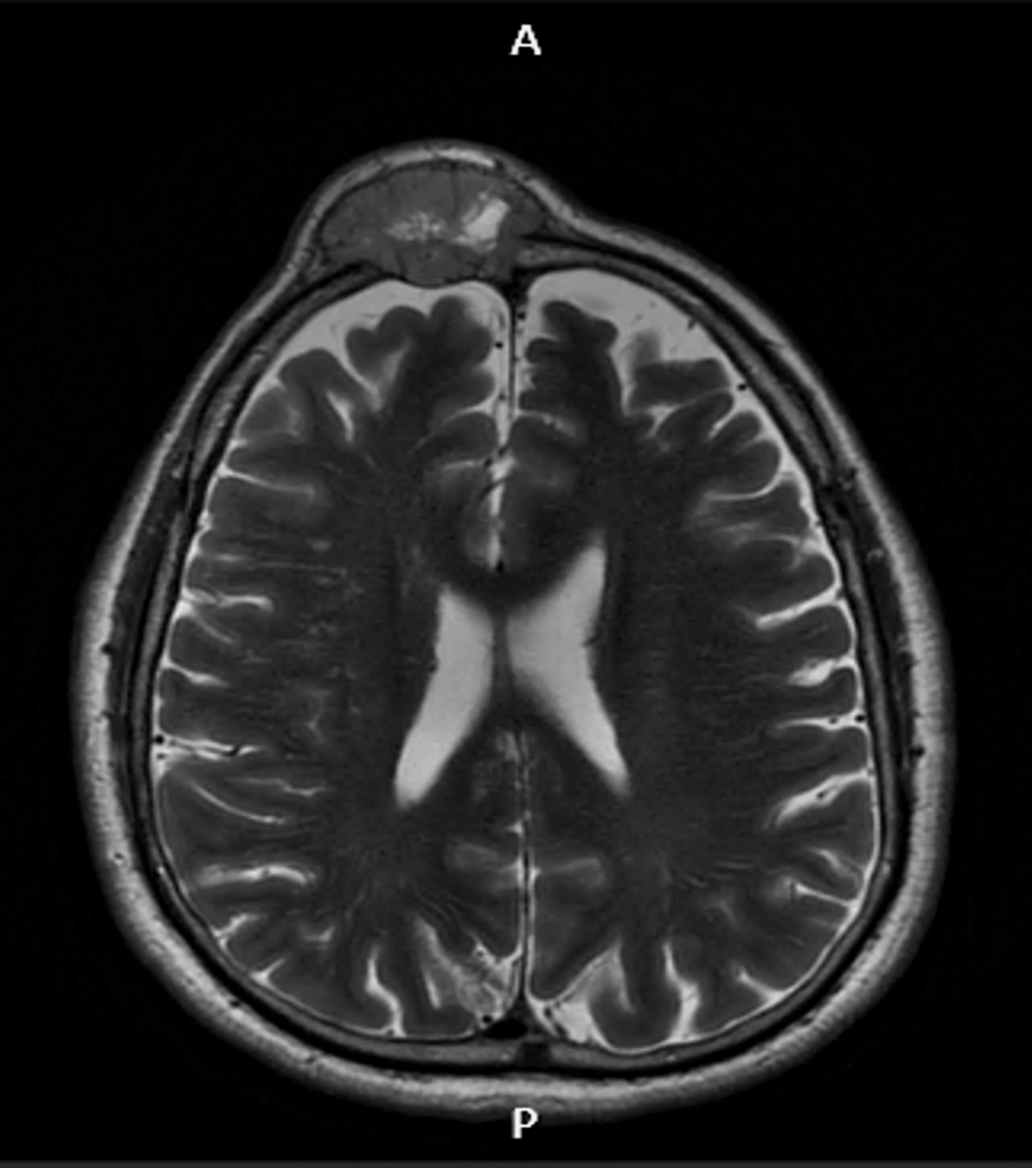
Cranioencephalic MRI in axial section The image shows an axial section in T1 fluid-attenuated inversion recovery (FLAIR).

**Figure 4:**
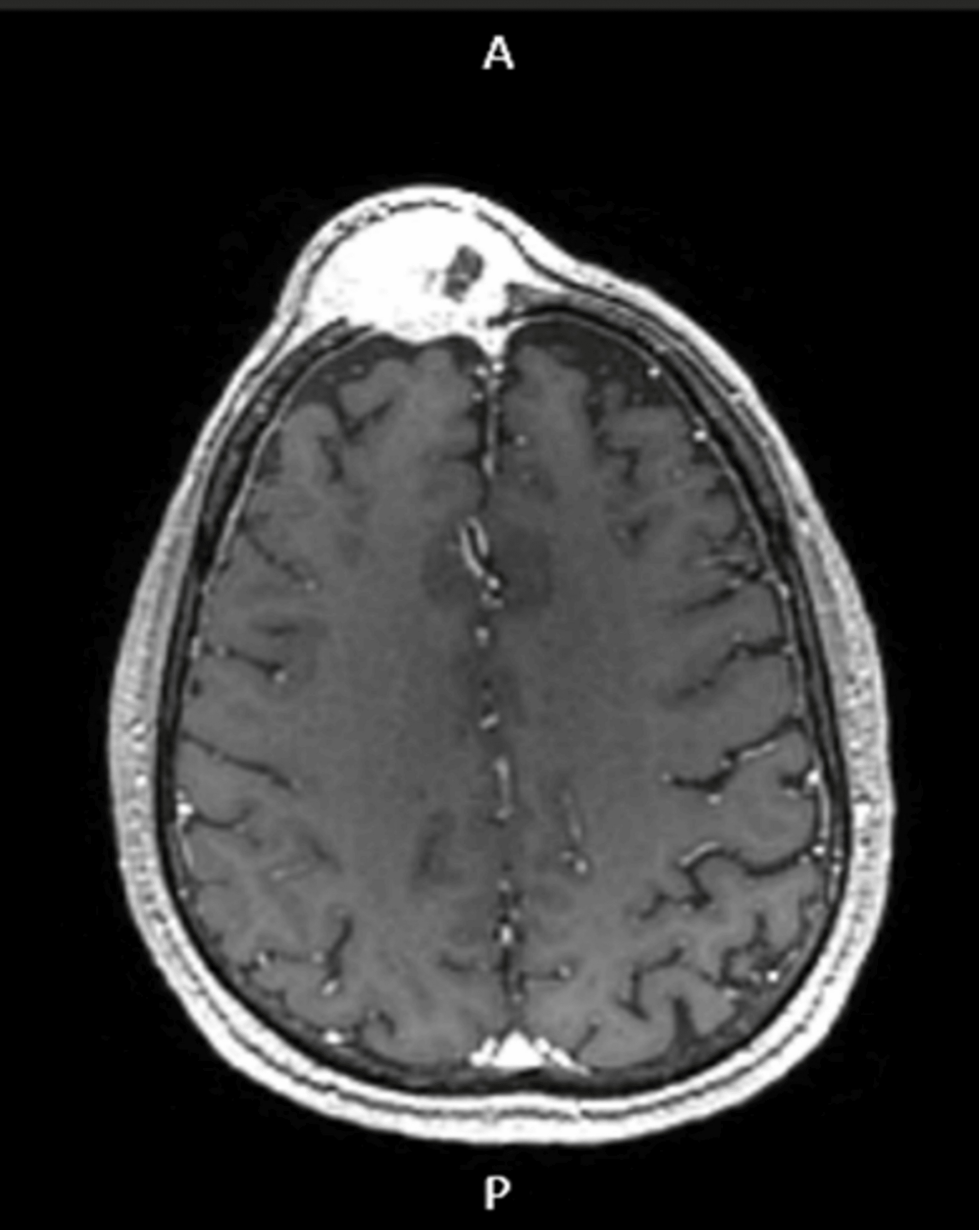
Cranioencephalic MRI in axial section The image shows an axial section in T1 gadolinium.

**Figure 5:**
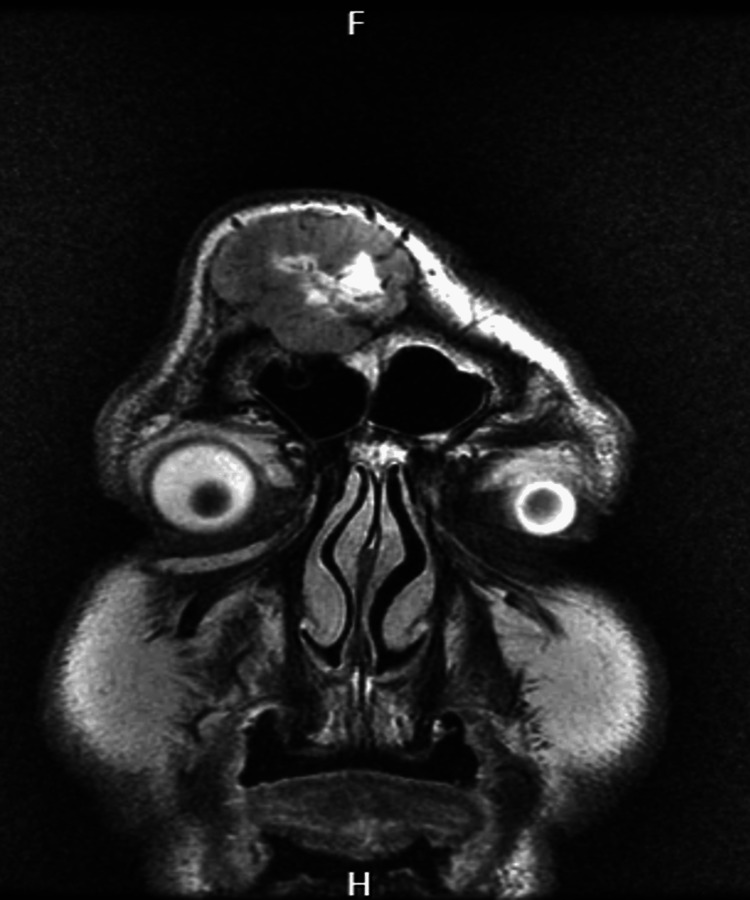
Cranioencephalic MRI in coronal section The image shows a coronal section in T2.

**Figure 6:**
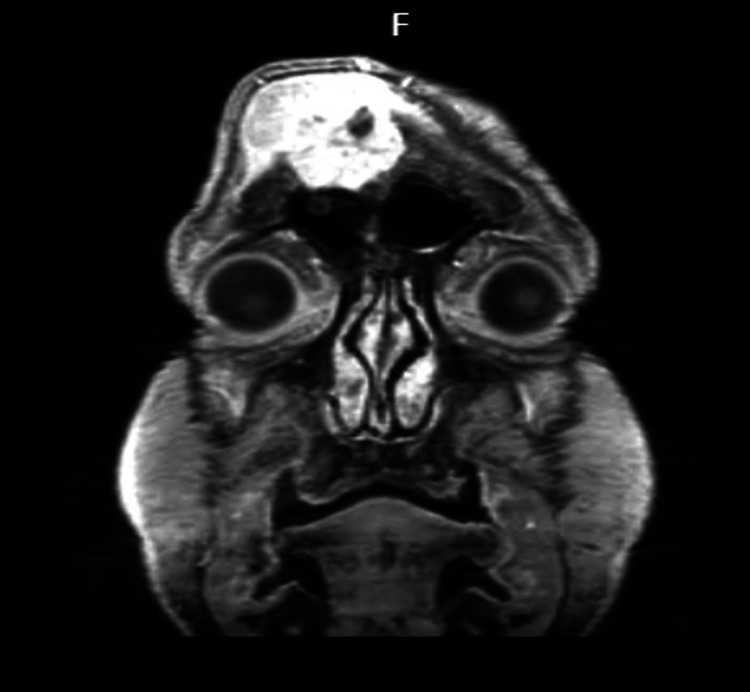
Cranioencephalic MRI in coronal section The image shows a coronal section in T1 gadolinium.

An urgent contrast-enhanced brain CT scan was carried out, which confirmed a single osteolytic lesion in the right anterior parasagittal frontal region with an expansile tissue component measuring 21x26 mm. It was associated with intracranial/dural invasion and was located near the superior longitudinal sinus. Given the patient's characteristics and age group, a primary versus a secondary tumor lesion was the most likely diagnosis. The patient also underwent a cranial MRI, which revealed no intraparenchymal brain lesions, as shown in the images below (Figures 1-6).

The patient was referred to the hospital specialties hematology-oncology and neurosurgery. Following the evaluation by neurosurgery, a thoraco-abdominopelvic CT scan was requested, which highlighted the presence of a mass in the right kidney in its middle/lower third, measuring 56x50x49 mm (TxAPxCC), consistent with RCC, and a small 6 mm nodule in the right middle lobe of the lung, single, with the possibility of either a primary or secondary pulmonary lesion. The remainder of the examination did not reveal other lesions.

**Figure 7:**
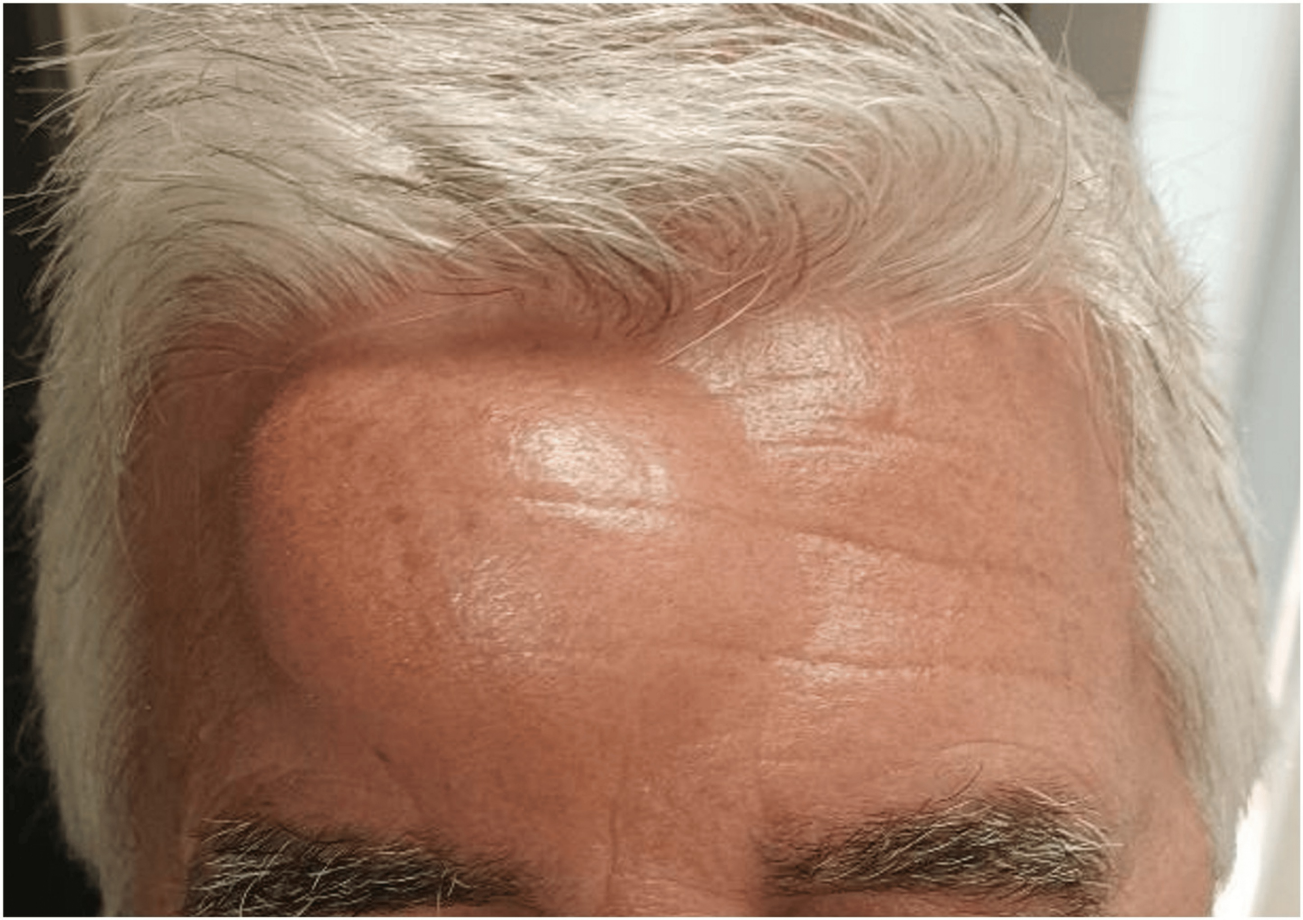
Frontal bone metastasis in frontal view The image shows the preoperative frontal bone metastasis of RCC in frontal view. A lesion of about 6 cm in diameter and 3 cm of protrusion is observed. RCC: renal cell carcinoma

**Figure 8:**
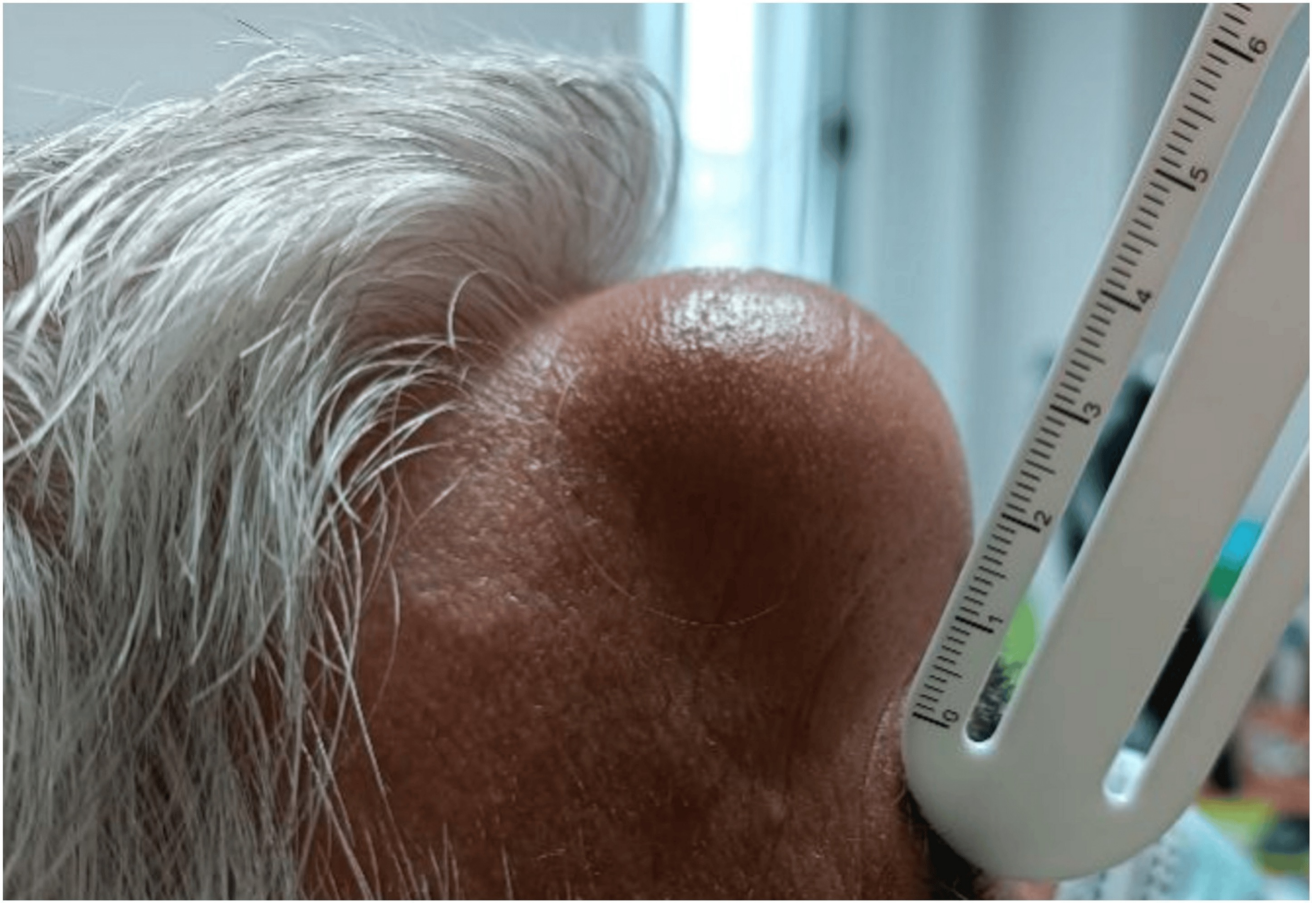
Frontal bone metastasis in the right lateral profile The image shows the preoperative frontal bone metastasis of RCC in the right lateral profile. A lesion of about 6 cm in diameter and 3 cm of protrusion is observed. RCC: renal cell carcinoma

In this context, the patient was referred to the urology consultation, where radical right nephrectomy was proposed, followed by surgical intervention for the metastatic frontal lesion. Before surgery, the lesion was approximately 6 cm in diameter and 3 cm in protrusion, as shown in Figures 7-8.

The histopathological results of the surgical specimens confirmed that the diagnosis was RCC, clear cell subtype, and secondary metastasis, respectively. Subsequently, due to the higher risk of recurrence, the patient underwent systemic therapy with pembrolizumab and axitinib, as well as cranial radiotherapy sessions.

Currently, the patient continues follow-ups with urology and is undergoing a course in systemic therapy in oncology. The latest thoraco-abdominopelvic and cranio-encephalic CT scans showed no new lesions. In addition to the hospital consultations, the patient has maintained follow-ups with his FD at the health center.

## Discussion

When not diagnosed accidentally, RCC can present with a variety of symptoms, which are generally related to the invasion of adjacent structures or distant metastasis [1-3]. The classic triad occurs in only 4-17% of cases [1]. Other signs and symptoms may be due to disease metastasis or paraneoplastic syndrome. Examples include fever, anorexia, weight loss, fatigue, cachexia, liver dysfunction, and involvement of the inferior vena cava with lower limb edema and right-sided varicocele [1-3,6]. Analytically, we may find anemia [6] or erythrocytosis [3], elevated alkaline phosphatase (21.1% in a study with a total of 365 patients) [7], increased lactate dehydrogenase [2], hypercalcemia [3,6], thrombocytosis [2], and secondary amyloidosis [6].

However, many patients are asymptomatic until an advanced stage of the disease [1,2]. Metastasis most commonly occurs in the lungs, bones, liver, and brain [1,8].

The major risk factors are advanced age, male, obesity, and smoking, with the last one being the most well-established modifiable risk [1-5]. Other identified risk factors include hypertension [1-3,5], regardless of antihypertensive medication, acquired cystic kidney disease [1,2,4], chronic kidney disease [1,2,4], occupational exposure to cadmium [1] and trichloroethylene [1,2,4], prolonged use of anti-inflammatory drugs [3,4], and family syndromes, with von Hippel-Lindau disease being the most common [1,2].

The prognosis of RCC can vary from months to several years, depending on clinical characteristics, histology, laboratory assessment, and radiographic findings, with the most critical factor being the anatomical extent of the disease in the tumor, node, metastasis (TNM) classification [2]. Therefore, early diagnosis is essential to improve the prognosis of these patients [1], as early-stage disease has better survival rates [2].

Given the subtle clinical presentation, this clinical case aims to highlight the need for high diagnostic suspicion.

As mentioned earlier, the patient initially presented to the clinic with a two-week history of swelling in the right frontal region, 3 cm in diameter, tender to palpation, and without inflammatory signs. The laboratory results were unremarkable. At first glance, the primary differential diagnoses were lipoma or sebaceous cysts in the frontal region. However, the contrast-enhanced brain CT revealed a tumor lesion. This clinical case illustrates the subtle clinical presentation often characteristic of RCC. Retrospectively, some "clues" in the patient's history could have pointed to this diagnosis, although it would have been challenging to consider at an early stage: male sex, knowing that this neoplasm is almost twice as common in men [1-4]; risk factors, including past smoking with a significant pack-year history, hypertension, and obesity [1-5]. However, many factors ruled out this diagnosis: unremarkable lab tests, absence of symptoms or signs that would raise suspicion, and the differential diagnosis with other more common conditions in the population.

The case presented reminds us of the characteristics of family medicine, as defined by the World Organization of Family Doctors (WONCA) since it is typically the first point of contact for patients with the healthcare system [9]. Thus, the FD should be aware of how to address modifiable risk factors, such as obesity, tobacco abuse, and hypertension, as seen in this case, and be capable of managing diseases that present in an undifferentiated manner at an early stage of their natural history, which may require urgent intervention [2,9]. Timely diagnosis and a multidisciplinary team approach were also crucial in improving the patient's prognosis.

## Conclusions

RCC accounts for the majority of cases of primary kidney neoplasms, many of which are asymptomatic until an advanced stage. Given the subtle clinical presentation of the disease, this clinical case discussion aimed to raise awareness of the diagnostic suspicion.

As the first point of contact for patients with the healthcare system, the FD plays a crucial role in managing risk factors associated with this pathology and in the early management of the disease, which often presents in a very undifferentiated form at the initial stage. Therefore, the work of a multidisciplinary team is essential, as is timely intervention, to ensure the success of these cases.
